# Draining the specialized nursing brains, the emigration paradigm of Ghana: A cross‐sectional study

**DOI:** 10.1002/nop2.1662

**Published:** 2023-02-23

**Authors:** Collins A. Poku, Abena K. Abebrese, Catherine K. Dwumfour, Agnes Okraku, Dorcas Acquah, Victoria Bam

**Affiliations:** ^1^ Department of Nursing Kwame Nkrumah University of Science and Technology Kumasi Ghana

**Keywords:** brain drain, emigration paradigm, specialist nurses, sub‐Saharan Africa

## Abstract

**Aim:**

The study aimed to determine the emigration intentions of specialist nurses (SNs) and ascertain the influencing factors, implications and mitigating factors in Ghana.

**Design:**

A cross‐sectional study.

**Methods:**

The sample was composed of 225 participants conveniently selected from a tertiary facility in Ghana. A turnover intention scale and the researchers' developed questionnaire were used to collect the data between June 1 and September 30, 2021. Data were analysed through descriptive statistics and linear regression.

**Results:**

The composite mean score for specialist nurses' intention to migrate was high (mean = 3.43); and the push factors accounted for the intentions explaining 48.6% of the variation (*R* ^2^ = 0.486, *F*_(5, 219)_ = 38.46, *p* < 0.001). The associated challenges of specialist nurses' emigration are increased cost of training new specialist nurses, poor quality of specialist nursing care, burnout syndrome among staff and poor patient health outcomes.

## 1 INTRODUCTION

The health workforce is one of the important pillars of the health system. A health workforce that addresses safe staffing levels (adequate quantity and skill mix) promotes the achievement of the sustainable development goals (SDGs) through better patient safety and nursing job outcomes (Saville et al., 2019; WHO, 2016a). Among the health workforce, the nursing workforce represents the largest individual group (Szabo et al., 2020), and specialist nurses (SNs) are key to advanced nursing activities. The SNs who are experts in the key fields of nursing practice and carry out tasks including ordering testing, diagnosing and administering simple treatments, perform their duties in a range of hospital settings and also collaborate with other professionals to provide the clinical team with high‐quality, patient‐centred care and support (Dossey et al., 2019; Miyamoto & Cook, 2019). The healthcare industry in the sub‐Saharan African (SSA) region is mostly characterized by inadequate human resources and a quality skill mix (Christmals & Armstrong, 2019; Karan et al., 2021). Among the elements that contribute to migration are the economic benefits, and the quest for better professional opportunities, among other reasons (Lowe & Chen, 2016; Sanou et al., 2014).

Determining the predictors of SNs migration will inform stakeholders of the mitigating factors to ameliorate the challenges with the phenomenon.

### 1.1 Background

In the last decade, the World Health Organization (WHO) has reported an increase of 60% emigration of the health workforce to higher income countries (HICs) (Szabo et al., 2020; WHO, 2018). This has been necessitated by the shortfall of the nursing workforce in HICs. For instance, the United States estimated about 550,000 nursing workforce shortfall in 2019 (Spurlock Jr, 2020). There is, therefore, a strong commitment by HICs to address the nursing shortage as the global shortage is projected to be 7.6 million by 2030 (Goh & Lopez, 2016; Shaffer et al., 2022; WHO, 2016b).

This explains the vigorous recruitment of the nursing workforce from low‐ and middle‐income countries (LMICs) to fill the gap, with the position well informed by the regulatory requirements for staffing in the health system in HICs (Efendi et al., 2017; Haddad et al., 2020). Moreover, the global ageing population and the surge in both communicable and noncommunicable diseases have been projected to result in considerable demand for the nursing workforce in HICs (Pung & Goh, 2017). It is expected that more nursing jobs will be available than all other professions in the next decade (Auerbach et al., 2015; Goodare, 2017) and with increased migration. The situation will impact negatively the healthcare systems globally (Salsberg & Martiniano, 2018; WHO, 2016b).

Migration is the movement of people across countries. Earlier research reports indicate that health professionals in SSA have been migrating particularly to the United States and the United Kingdom since the inception of medical education (Adovor et al., 2021; Ngoubene‐Atioky et al., 2021). In the United Kingdom, the National Health Service (NHS) alone estimated 3395 health workforce of Ghanaian descent; an increase from the past figure of 3236 (Baker, 2018).

Shaffer et al. (2022) reiterate that migration is an effective developmental tool to solve unemployment problems, reduce poverty and mobilize financial resources for growth. Increased flows of remittances, in particular, have also been used to justify the benefits of emigration to LMICs. In 2019 for instance, it was estimated that migration contributed over US$4 billion in remittances to Ghana contributing 6.05% to the GDP (Teye, 2022). Migration has also been argued to facilitate and contribute to both social and economic advancement but its negative effect has always outshone the benefits to LMICs.

In LMICs, it is considered a loss of intellectual and technical personnel, and it affects the workforce distribution (Ayalew et al., 2021; Chand, 2019; Stokes & Iskander, 2021). This one‐way movement of highly skilled workforces largely profits the host countries, as it depletes access to specialized care from SNs and, therefore, poses a major problem to the health sector of LMICs. Earlier reviews on the emigration of the nursing workforce showed a negative impact on the quality of nursing practice, and it is likely to continue for years to come (Chand, 2019; Mlambo & Adetiba, 2019; Okafor & Chimereze, 2020). With the key role of the nursing workforce in achieving the SDGs and Universal Health Coverage (UHC) (WHO, 2020), the recent migration paradigm of SNs possesses major potential health security and hazards to less developed health systems, and thus threatens the advances that have been made in the achievement of the health‐related SDGs (National Academies of Sciences & Medicine, 2016; United Nations, 2015).

Not only does the emigration of SNs compromise the health systems in the LMICs but it also carries with it, significant financial losses from the investment made in educating health professionals (Adesote & Osunkoya, 2018; Atte, 2021; Ekanayake & Amirthalingam, 2020). Studies have shown that HICs, such as Australia, Canada, the United Kingdom, the United States, the United Arab Emirates and other European countries, have profited from the migration of the nursing workforce, whereas LMICs lose huge investments in human capital (Chimenya, 2018; Yeates, 2010). On the contrary, it is important to state that there is a win‐win situation for countries with formalized migration arrangements with the HICs (Cabanda, 2017; Ortiga, 2014). The situation in Ghana is not well articulated and communicated through migration policy.

There has also been a flurry of scholarly investigations focused on exploring the rationale behind the increased emigration of the health workforce from SSA countries. Studies across different settings have documented push and pull factors that account for the observed pattern of the emigration of the health workforce including poor working conditions such as long working hours (Humphries et al., 2015), career development‐related factors (Mlambo & Adetiba, 2019), inadequate resources and increased workload (Yuksekdag, 2018). Significantly, most nurses are attracted to HICs by the prospect of better remuneration (Okeke, 2014). For instance, it is enough justification for nurses in SSA who receive as low as one‐eighth of what their counterpart receives in the United States to migrate (Edwards et al., 2021). Other factors that have also been cited include individual‐related factors such as improvement in social status and lifestyle (Dohlman et al., 2019; Karaduman & Çoban, 2019) and political instability (Adeyemi et al., 2018).

In SSA, nursing workforce migration has been attributed to inadequate logistics and workplace safety concerns, increased workload, low salary, and poor or lack of benefits, lack of career, technology, and specialization advancement, among other negative working conditions (Chimenya, 2018; Salami et al., 2014). The migration push and pull factors constitute a well‐established atmosphere in LMICs where the migration of health professionals is not only encouraged but adored as some mentors become elated when their mentees successfully migrate to high‐resourced countries for greener pastures (Adovor et al., 2021; Chand, 2019).

Moreover, the International Council of Nurses (ICN) has claimed that the COVID‐19 pandemic has caused burnout, and consequently increase the attrition rate of nurses in most HICs. This is evidenced by most healthcare facilities seeing a 19% rise in nursing turnover since the pandemic began in March 2020; an increase of over 3%. An estimated 25% of nurses also have the intention to quit their job due to constraints from the pandemic. Again, more than a 1000 nurses have died as a result of the epidemic with the ICN confirming that the profession is experiencing major trauma, especially in HICs (Berlin et al., 2021; ICN, 2020; Shaffer et al., 2022).

It has been asserted that the health human resource crisis characterized by a severe migration of the specialized nursing workforce in Ghana is partly due to uncorrected implementation lapses of certain policies, and this has resulted in a persistent shortage of SNs in healthcare facilities. Even though, thousands of newly qualified nurses remain unemployed (Asamani et al., 2020), employing them cannot wholly solve the problem of the loss of these highly knowledgeable, skilled, and experienced cadre of nursing workforce (SNs), and the resources used for their training. Some health system structural failures within the country such as the lack of a well‐defined career progression for SNs foster the emigration paradigm. The lapse though not wholly the cause of emigration, serves as a contributing factor. There is also an assumption by many of the increase in the brain‐drain paradigm from an enticing remuneration from the pull factors in the host countries (Hashish & Ashour, 2020). There seems to be no implementation plan for promoting the migration of nurses, especially concerning education and bilateral cooperation; if there is, then the process and its impact remain unclear.

Nursing workforce emigration in Ghana appears gloomy and depressing with low staff morale, and serious nurse specialist workforce shortages, among other factors (Asabir, 2018; Teye, 2022). The constraints on public sector jobs combined with nurses' propensity to emigrate over the past decade have led to a low specialist nurse‐to‐population ratio. A previous Ghana Health Service study shows that Ghana's nursing staff‐to‐population ratio was 1:959 in 2014 and varied from 1:2173 to 1:959 from 2007 to 2015 (Asiedu et al., 2018).

The resolution of emigration challenges requires all stakeholders to eschew the rhetoric and be flexible and proactive to enact policies that would improve welfare and reduce the rates among nurses (Aniche, 2020; Clemens et al., 2019; Efendi et al., 2021).

Although studies on the factors that contribute to nurses' migration have been established in certain countries, no published data exist concerning the influencing factors that inform the migration of SNs from Ghana. Adequate validated data for policy formulation that will use the benefits of emigration on socio‐economic development is also lacking. From anecdotal evidence, many SNs are migrating from Ghana with many others nurturing the intention to migrate. The labour mobility and socio‐demographic studies have, however, shown that personal variables, work experience, social groups, and links to migration networks crucially determine migration decisions (Rizwan et al., 2022). It is widely recognized that understanding the motivating causes and the objectives of the various migratory actors, particularly emigrants, can suggest potential points of contact or stakeholders when formulating migration policy (Teye, 2022). The findings of the study may also give data on multiple actions needed at various policy levels in both Ghana and partner‐receiving countries to moderate the negative effects of the emigration of the nursing workforce, and also help reduce the increased turnover of the SNs in the health sector.

The study, therefore, sought to assess the push and pull factors associated with the Ghanaian SNs' intention to migrate. It also examined the implications of emigration on healthcare and the mitigating factors for the challenge.

### 1.2 Research questions

- What are the emigration intentions of SNs?
- What are the implications for the emigration of SNs?
- What are the mitigating factors for the emigration of SNs?
- What are the predictors of the emigration intentions of SNs?

## 2 METHODS

### 2.1 Design

A cross‐sectional descriptive design was used for the study. This design was chosen because it could best assess the emigration intentions of SNs, the push and pull factors influencing the emigration, its implication on the nursing profession and the mitigating factors for it.

### 2.2 Setting and samples

The study was conducted at the second biggest government‐owned Teaching Hospital in Ghana. The facility is a 1200‐bed capacity hospital with 10 clinical directorates including Anaesthesia and Intensive Care Unit, Child Health, Dental, Eye, Ear, Nose and Throat (DEENT), Diagnostics, Medicine, Obstetrics and Gynaecology, Oncology, Poly Clinic, Accident and Emergency and Surgery. The hospital receives referrals and specialist cases from other parts of the country and neighbouring countries. The health workforce is made up of physicians, nurses, pharmacists, laboratory technicians, etc. whose services help in the smooth running of the hospital; and all employees are paid by the government. The hospital employs all cadre of nursing staff and reflects the experiences and the general status of nursing in Ghana. The SNs provide services on Ear, Nose, and Throat (ENT), ophthalmic, emergency, critical and peri‐operative, oncology, anaesthesia, public health and paediatric, among others. The study had 225 fully answered questionnaires returned (response rate of 70.3%). Questionnaires that were not filled out completely were excluded from the analysis.

### 2.3 Study participants

The study was carried out among SNs at a Teaching Hospital in Ghana between June and September 2021. The nursing workforce of the facility is estimated to be approximately 4248 with an average age of 30 years. The SNs (ophthalmic, peri‐operative and critical, emergency, public health, oncology, palliative, ear, nose and throat, psychiatric, and paediatric nurses) account for approximately 1150 of the nursing workforce. Although an average of 22,000 nurses and midwives pass out every year, the percentage of SNs is not encouraging. The exact statistics of nurses who have migrated in recent years are not readily available, but there are reports of an alarming rate in the Ghanaian health sector, as an estimated 24% Ghanaian trained nurses are working in HICs (Asamani et al., 2020; Preko et al., 2019).

### 2.4 Sampling technique and sample size

The participants for the study were sampled through a convenience sampling method. Krejcie and Morgan's sample determination formula (Krejcie & Morgan, 1970) was used to estimate a sample size of 291 participants. Ten percent of the sample (approx. 29 participants) was added to allow for possible attrition. Thus, we aimed to recruit a total of 320 for this study.

### 2.5 Measurement

#### 2.5.1 Push and pull factors for nurses' emigration

A researcher‐developed questionnaire based on an extensive review of related literature (Gea‐Caballero et al., 2019; Goštautaitė et al., 2018; Mobley et al., 1978) was used to elicit the pull and push factors, implications and mitigating factors for nurses' emigration. The questionnaire was revised to remove ambiguity after pretesting it on SNs at another hospital in a similar environment. The push and push factors comprised 23 items: economic factors (3 items), health system factors (6 items), professional factors (6 items), social factors (5 items), political factors (3 items); and multiple responses on the implications (1 item) and mitigating factors (1 item) for the migration of SNs. The questions were scored on a 5‐point Likert scale; 1 = strongly disagree to 5 = strongly agree. The composite mean score of each sub‐scale was grouped as low (<2.5) or high (≥2.5). The Cronbach's alpha coefficient of the tool after pre‐testing was 0.85.

#### 2.5.2 Emigration intentions of specialized nurses

A three‐item turnover intention scale designed by Mobley et al. (1978) was used to measure SNs' emigration intention. The items included ‘I often think of leaving the organization, ‘I intend to look for a new job within the next year’, and ‘If I could choose again, I would not work for this organization’. The items were measured on a five‐point scale; from 1 = “Strongly disagree” to 5 = “Strongly agree”. A composite mean score above 2.5 indicated higher emigration intentions. The internal consistency coefficients for the scale in other studies ranged between 0.80 and 0.90 (Labrague et al., 2020; Laeeque et al., 2018).

### 2.6 Data collection method

Following Research Ethics Committee approvals, the participants were reached through the Director of Nursing Service. The purpose of the study, its risk, and benefits, the rights and the procedure involved, were explained to all participants. Participants who voluntarily consented to be part of the study were sampled through convenience sampling and questionnaires were administered to them.

### 2.7 Data analysis

The results were analysed using the IBM SPSS Statistics Version 25. Descriptive analyses such as composite mean score and standard deviation were used to explore the perception of SNs on emigration intentions and push factors accounting for emigration, while the frequencies and percentages were used for ranking the implications and the mitigating factors of nurse emigration in healthcare. The multivariate analysis was also done using linear regression to test the predictive effects of the push factors on the emigration intention of SNs. Suitable assumptions of regression analysis were examined and the data fulfilled the assumptions for no multicollinearity and independent errors (Durbin–Watson = 2.02). A Scatter plot was also used to confirm the assumptions of linearity and homogeneity. A value of *p <* 0.05 was set as the level of statistical significance.

### 2.8 Ethical consideration

The study was carried out subject to approval by the management of the study site and ethical clearance sought from the Institutional Review Board of REDACTED. Permission was sought and granted by all individuals who were included in the study. Participants' anonymity was maintained and informed consent was also sought before the questionnaire was administered.

## 3 RESULTS

### 3.1 Socio‐demographic data of participants

More than 70% (*n* = 160) of the participants were females; 47.6% (*n* = 107) had attained a Bachelor of Science, 73.3% (*n* = 165) were aged between 31 and 40 years and 70.7% (*n* = 159) were married. More than half of the participants (51.1%, *n* = 115) had worked between 6 and 10 years, while close to a fifth (21.8%, *n* = 49) were specialists in Emergency Nursing as shown in Table 1.

**TABLE 1:** Socio‐demographic characteristics of participants.

Variable	Categories	*N* = 225 (%)
Gender	Male	65 (28.9)
	Female	160 (71.1)
Last educational	Diploma	101 (44.9)
Status	Bachelor's degree	107 (47.6)
	Postgraduate degree	17 (7.6)
Age	30 years or less	49 (21.8)
	31–40 years	165 (73.3)
	41–60 years	11 (4.9)
Marital status	Single	66 (29.3)
	Married	159 (70.7)
Working experience	<5 years	50 (22.2)
	6–10 years	115 (51.1)
	11–20 years	55 (24.5)
	21–30 years	5 (2.2)
Specialty	Critical Care and peri‐operative Nurse	28 (12.4)
	Emergency Nurse	49 (21.8)
	ENT Nurse	17 (7.6)
	Oncology nurse	5 (2.2)
	Ophthalmic Nurse	19 (8.4)
	Paediatric Nurse	38 (16.9)
	Public Health Nurse	4 (1.8)
	Mental Health Nurse	45 (20.0)
	Nurse Anaesthetist	20 (8.9)

*Source*: Field Data, 2021.

### 3.2 Emigration intentions and factors that influence the emigration of specialized nurses

As shown in Table 2, the emigration intentions and the push factors account for the increased emigration of SNs. The composite mean score for SNs' intention to emigrate to high‐resourced countries was high (M = 3.43, SD = 0.72). Of the 194 SNs who reported on their emigration intentions, nearly half (45.3%, *n* = 88) indicated North America as the preferred destination.

**TABLE 2:** Emigration intentions of specialized nurses and the push factors.

Items	M	SD	Min	Max
Emigration intention	**3.43**	**0.72**	**1**	**5**
Overall push factors	3.09	0.89	1	5
Economic factors	3.84	0.81	1	5
Poor salaries	3.81	0.83	1	5
De‐evaluation of the currency	3.79	0.75	1	5
Poor cost of living	3.93	0.85	1	5
Health System factors	3.57	0.86	1	5
Poor working condition	3.33	0.68	1	5
Work overload	3.56	0.87	1	5
Poor health infrastructure	3.36	0.70	1	5
Inadequate equipment	3.63	0.90	1	5
Poor management and supervision	3.68	0.99	1	5
Active recruitment overseas	3.83	1.02	1	5
Social factors	3.28	1.02	1	5
Offer of better quality of life	3.56	0.91	1	5
Access to social network	3.69	1.01	1	5
To join family	3.33	0.97	1	5
Desire to experience different work environment	2.89	1.00	1	5
Desire to gain foreign citizenship	2.94	1.23	1	5
Political factors	1.63	0.68	1	5
Safety and security reasons	1.12	0.49	1	5
Government mismanagement	1.72	0.69	1	5
Influence of colonial connections	2.05	0.86	1	5
Professional factors	3.12	1.06	1	5
Lack of career advancement	3.28	1.08	1	5
Lack of promotion	2.08	1.11	1	5
Low professional satisfaction	3.16	0.87	1	5
Opportunity to gain better clinical experience	3.39	1.05	1	5
Desire to gain foreign professional qualification	3.25	1.12	1	5
Opportunity for professional networking	3.56	1.15	1	5
Preferred destination	Frequency (%)			
North America	88 (45.3)			
Europe	63 (32.5)			
Australia	32 (16.5)			
Others	11 (5.7)			

The study also revealed SNs perceived push factors as influencing their emigration with a composite mean score (3.09 ± 0.89). Economic factors and health system factors had the highest composite mean score of 3.84 (SD = 0.81) and 3.57 (SD = 0.86) respectively. Political factors, however, had a low mean score of 1.63 (SD = 0.68). The detailed push factors have been presented in Table 2.

### 3.3 Implications of specialized Nurses' emigration on healthcare

Table 3 shows the implications of the emigration of SNs to healthcare as identified by participants. A greater number of SNs (*n* = 195, 86.7%) stated that the major implication is the increased cost of training new SNs while 131 (58.2%) of the participants indicated increased mortality as a consequence of SN emigration.

**TABLE 3:** Implications of migration of Specialized Nurses to healthcare.

Implications of emigration of nurses	*N* = 225 (%)
Increased cost of training new specialist nurses	195 (86.7)
Decreased quality of care	188 (83.6)
Burnout of nurses	182 (80.9)
Shortage of personnel	176 (78.2)
Increased workload	171 (76.0)
Overcrowding	170 (75.6)
Poor patient health outcome	168 (74.7)
Patient dissatisfaction	158 (70.2)
Prolong hospital stay	152 (67.6)
Increased mortality	131 (58.2)

*Source*: Field Data, 2021.

### 3.4 Mitigating factors

From Table 4, the participants generally perceived all the seven listed items as mitigating factors to combat the emigration of SNs with all items scoring >50%. Most of the SNs perceived an improved pay/salary that is commensurate with the workload in the healthcare setting as the highest mitigating factor for the emigration of SNs. They, however, perceived enhanced collaboration and teamwork as being the relatively least important mitigating tool on the emigration paradigm in Ghana.

**TABLE 4:** Mitigating factors for the emigration of specialized nurses in Ghana.

Mitigating factors	*N* = 225 (%)
Improved pay/salary to commensurate with workload	196 (87.1)
Effective nursing leadership/good governance	192 (85.3)
Professional practice environment	183 (81.3)
Adequacy of material resources for working	178 (79.1)
Fair treatment from nurse managers	175 (77.8)
Easy access to continuous professional development	169 (75.1)
Enhanced collaboration and teamwork	140 (62.2)

### 3.5 The predictors of push factors on emigration intentions of specialized nurses

Table 5 shows the statistical analyses to test the predictors of emigration intentions by SNs. In the model, all the variables together predicted the emigration intention of SNs explaining 48.6% of the variation (*R* ^2^ = 0.486, *F*_(5, 219)_ = 38.456, *p* < 0.001). However, the emigration intentions were significantly predicted by the push factors including economic factors (*β* = 0.361; *p* < 0.001), health system factors (*β* = 0.250; *p* < 0.001), and social factors (*β* = 0.133; *p* < 0.05). The political and professional factors did not significantly contribute to the model. In effect, a unit increase in the score of health system factors was associated with an increased migration intention of SNs by 0.250 points. Similarly, an increase in economic factors by 0.361 was noted for a unit of increase in the score of emigration intention. Furthermore, an increase in social factors (0.133 points) was noted for a unit increase in the score of emigration intention of SNs.

**TABLE 5:** Linear regression model for predictors of emigration intention by specialized nurses.

	B	SE	*β*	*t*	Sig.
(Constant)	0.302	0.077		3.944	0.000
Health system factors	0.020	0.005	0.250	3.862	0.000
Professional factors	0.003	0.006	0.035	0.566	0.572
Economic factors	0.032	0.006	0.361	5.013	0.000
Political factors	0.008	0.006	0.085	1.265	0.207
Social factors	0.025	0.011	0.133	2.307	0.022

*Note*: *R* ^2^ = 48.6, *F*_(5, 219)_ = 38.456, *p* < 0.001.

Dependent variable: emigration intentions, 95% confidence level (*α* = 0.05).

Unstandardized beta (B) is used to denote sample regression coefficient and interprets the effect of each predictor on the outcome; Standard beta coefficient (β) is used to denote coefficient in population and explains the unique contribution of the predictors to the outcome variable; SE, standard error explaining how well a regression model fits a data set.

## 4 DISCUSSION

The study examined the push/pull factors that account for the migration of SNs from Ghana. The study showed that most SNs are influenced by the economic, health system and social factors for their emigration intention. Similar to the findings of this study are Jenkins (2016) and Komušanac (2021) who reported that the emigration of health professionals stems from the multifaceted push and/or pull factors. They posited that SNs' motivation to migrate is not limited to a single reason, but economic reasons, professional development, social concerns, health system and political factors. Agreeing with the findings, Prescott and Nichter (2014) also reported that the emigration of SNs is mostly influenced by pull or push economic logic. Ostensibly, impoverished socio‐economic climate (poor salary structure regarding work dynamics, penury living conditions, currency devaluation, joining family/friends, desire to experience working in a different environment), low professional satisfaction and fulfilment and the influence of colonial connections have also been pointed out as possible causes of migration in Ghana; and this position is supported by Johnson et al. (2014) and Walton‐Roberts et al. (2017).

The study reports that SNs' migration is largely due to push factors such as economic challenges (low average salary) and pull factors including higher wages, improved quality of life, growing economy and prestigious educational opportunities (Dywili et al., 2013; Lanati & Thiele, 2021; Marć et al., 2019; Nortvedt et al., 2020). Per the findings of this study, SNs are keen to take any opportune moment to travel to HICs for better remunerations because emigration is considered a human capital flight for nurses who desire to seek a higher standard of living due to favourable professional opportunities This is consistent with the study findings of Hashish and Ashour (2020), Abdel‐All et al. (2019) and Li et al. (2020). In the case of Ghana, there is a recruitment challenge as newly qualified nurses stay out of the job for a year or more due to the government's inability to employ them. This compounds the economic challenges of nurses (Asamani et al., 2020). Meanwhile, the experience and quality of most of these new nurses do not meet what the receiving countries required to plan formalized migration. The above situation is a sharp contrast to the happening in most HICs, where there is a shortage of nursing staff (Drennan & Ross, 2019; Haas et al., 2020; Marć et al., 2019).

Again, akin to the study findings, social factors were considered essential for migration and it is consistent with the position of Goštautaitė et al. (2018) and Nortvedt et al. (2020) who conclude that support from family and migratory networks in the country of destination are important elements conducive to nurses' migration.

The migration of SNs has a negative impact on the nursing profession such that it decreases the skilled and more experienced specialized nursing workforce while increasing the workload. This is consistent with the findings of Nicholas (2019), Olorunfemi et al. (2020) and Peters et al. (2020) who posited that emigration leads to unequal distribution of SNs. The situation results in poor quality nursing care delivery confirming the findings of Anetoh and Onwudinjo (2020) and Żuk et al. (2019). Again, Peters et al. (2020) recognized that in situations when nurses overwork, burnout syndrome is imminent, and negatively affects their relationship with colleagues and patients, which in turn has an impact on overall patient safety and care management. Supporting the findings of Griffiths et al. (2019) and Musy et al. (2021) on how a low nurse–patient ratio results in lower mortalities, this study found that emigration leads to a shortage of SNs that results in a prolonged stay in the hospital, poor patient health outcomes and increased mortality.

The current study concludes that mitigating factors should concentrate on nurse leaders establishing and maintaining a professional practice environment where good nursing governance and leadership will enhance work engagement and other nursing job outcomes, and this is consistent with the findings of Onu et al. (2021). For instance, most nurses have reported in the past as seeing their profession to be subservient to other professions in the healthcare industry; and have quit the profession due to the lack of autonomy through migration to high‐resourced countries (Edwards et al., 2021). As a way of empowering itself, the nursing profession should wean itself and promote good governance by building strong professional policies and regulations to advocate for the rights of its members. Additionally, the findings highlight that nursing workforce retention hinges on the recognized values placed on the profession through leadership support and an empowering practice environment. These measures and others, including expanding the capacity and output of healthcare facilities as reported by Hancock (2020) and Masanjala (2018) have been recommended in past studies to overcome the challenge of migration and eventually improve the specialized nurse–patient ratio in the country. Again, the desire for continuous professional development as highlighted in the study findings can be improved as it has been noted to promote the retention of SNs (Aravossitas & Sugiman, 2019; Chand, 2019). Moreover, creating a conducive practice environment in LMICs can go a long way in reducing the brain drain of skilled SNs from the country. Thus, career pathway policies centred on enticing SNs and espousing strategies to improve their well‐being and the aforementioned motivation should be developed to help reduce the phenomenon (Hashish & Ashour, 2020; Okafor & Chimereze, 2020).

The study findings stressed the need to improve the wages of nurses. This position agrees with Hashish and Ashour (2020) who advocated for healthcare managers to holistically ensure the role of managing both wage and non‐wage strategies in reducing the migration of skilled SNs. Although improvement of the salary structure for the nursing workforce alone cannot resolve the nursing brain drain challenge, it can help lessen it (Liu et al., 2022; Masanjala, 2018).

Although in most LMICs, enough nursing workforce is trained annually; the value of losing experienced SNs through migration cannot be solved by replacing them with less experienced newly trained nurses. Notwithstanding, respective government recruitment agencies should facilitate the employment of newly qualified nurses to reduce their waiting time. This will give hope for the nursing workforce who are willing to stay in the country to work (Onu et al., 2021). The findings of the study support the position of providing adequate material resources to support the sustenance of the health systems of the government as a motivation to reduce the frustration of improvising the care provided to the patient (Dieleman et al., 2019).

Given the benefits of migration of nursing workforce as reported in India and the Philippines, it will be profitable for SSA countries with training capacity to deliberately produce and promote nurses for “export” to destination countries like the United Kingdom and the United States to respond to global market demands (Cabanda, 2017; Ortiga, 2021). This means migrant‐sending states should develop policies that will make nursing training institutions adjust their curriculum to cater to foreign employers' needs and also strategies in facilitating the emigration of nurses. Since most of the destination countries prefer more advanced and highly SNs, countries should consider a two‐way approach training system; one for domestic use, and the other for the international market. Again, training of lower cadres such as certificate and diploma nurses should be upgraded to degree awarding and specialist programmes.

### 4.1 Limitations

This study was conducted among SNs in a Teaching Hospital in Ghana. Findings can, therefore, be generalized to a similar category of nurses. The information obtained from this study was subjective since participants only gave an account of their intention where there was no means to authenticate it; there may, therefore, be participants' bias. Detailing emigration and or emigration intentions, for instance, may have used other forms of data collection processes, such as interviewing nurses about their intentions or review of human resource data of the study settings in the past years. The study findings, however, were harmonious with the existing literature. Again, the scale was self‐developed after reviewing related literature, which may limit its validity and reliability; however, the Cronbach alpha after the pre‐test showed a satisfactory value.

## 5 CONCLUSION

Emigration of the nursing workforce in LMICs is in full force; policy‐makers in nursing have been provided with the needed information through this study to improve nursing workforce management to reduce brain drain. Development assistance, particularly concerning job creation in migrant source regions, is one of the key strategies advocated to address these root causes of irregular movement. Other methods to lower the rate of emigration have been suggested, such as promoting good governance (Knoll & de Weijer, 2016). This could be achieved through improved remuneration and career and professional growth for the nursing workforce. It is, therefore, recommended for both private and public healthcare managers to develop incentive packages such as hourly rate wages, and improved conditions of service for the nursing workforce to serve as motivating factors for retention. Even though ensuring strict adherence to the clearance and verification process by all stakeholders in the healthcare industry will allow all nurses, especially the most skilled and experienced ones, to duly oblige to their bonding years, keeping qualified nurses out of the job for years will result in higher intention to migrate. It will, therefore, be more prudent to recruit newly qualified nurses who benefited from government scholarships during their training in government healthcare facilities to improve the nurse–patient ratio and also enhance quality healthcare to the client. Although there is a significant financial reward from remittances, which account for a sizable portion of the national gross domestic products (GDPs) of the majority of source countries, stakeholders should make sure they preserve viable skilled staff to promote quality care delivery (Ratha et al., 2020; Shaffer et al., 2022). Conjointly, governments should work to include the viewpoints of migrants in migration policies given that micro‐narratives of migrants generally impact migration decisions. Although the outlined factors can help reduce the pace of emigration and/ or promote fair migration, the quality of specialized nursing care can be compromised if the phenomenon is not fully addressed.

The framework of bilateral collaboration in managing migration is provided by the WHO Global Code of Practice for the International Recruitment of Workforce encourages member governments to enter into bilateral agreements to control health professional migration in addition to providing recommendations for the ethical recruitment of health professionals from LMICs. The code also encourages policymakers to focus on bilateral agreements that necessitate the receiving countries to invest in the training and development of the health workforce (Cabanda, 2017; Ortiga, 2021; Thompson & Walton‐Roberts, 2019).

## AUTHOR CONTRIBUTIONS

CAP, AO, CKD and DA conceptualized and designed the study method. CAP, AO, DA and AKA carried out the data collection, analysis and interpretation of data. CAP, AKA, CKD and VB originally drafted the manuscript. All authors read, revised and approved the final manuscript for submission.

## FUNDING INFORMATION

No funding was received for this study.

## ETHICS APPROVAL AND CONSENT TO PARTICIPATE

Research Ethics Committee approval was given by the Komfo Anokye Teaching Hospital Institutional Review Board (KATH‐RD/CR21/126). Participants also gave written consent before data collection.

## CONSENT FOR PUBLICATION

All authors have approved the manuscript for submission.

## CONFLICT OF INTEREST STATEMENT

The authors declare that they have no competing interests.

## Data Availability

The data that supports the findings of this study are available in the supplementary material of this article.
